# Complexity of Secretory Chemokines in Human Intestinal Organoid Cultures Ex Vivo

**DOI:** 10.1016/j.gastha.2022.02.009

**Published:** 2022-04-08

**Authors:** C. COTTLE, M. ANBAZHAGAN, A. LIPAT, M. PATEL, A. P. PORTER, K. HOGAN, D. RAJAN, J. D. MATTHEWS, S. KUGATHASAN, R. CHINNADURAI

**Affiliations:** 1 Department of Biomedical Sciences, Mercer University School of Medicine, Savannah, Georgia; 2 Division of Pediatric Gastroenterology, Department of Pediatrics, Emory University School of Medicine & Children’s Healthcare of Atlanta, Atlanta, Georgia

Crohn’s disease (CD) is characterized by chronic inflammation of the mucosa, which involves the release of cytokines and chemokines that promotes further activation and infiltration of leukocytes.^1^ Leukocyte trafficking to the gut is mediated by the interaction of chemokines with G-protein-coupled receptors, and hence, this interaction can be therapeutically targeted to control mucosal inflammation.^2^ Despite this therapeutic potential, clinical trials have yet to show efficacy in chemokine-blocking intervention for CD management.^3^ For example, CCL25 recruits CCR9-expressing leukocytes, and blocking this interaction in a phase III clinical trial with Vercirnon was shown to be ineffective in the treatment of moderate to severe CD.^4^ This suggests the involvement of more than one chemokine that needs to be targeted in CD management, and the secretory chemokines of intestinal epithelium are unknown. In a recent report, we established an experimental protocol for defining the epithelial secretome in conditioned media of intestinal organoids derived from mucosal biopsies of a pediatric population and showed several interleukins, growth factors, and cytokines released from these cells.^5^ In the present study, we have extended those findings using the previous technique on non-inflammatory bowel disease (IBD) and CD pediatric patient-derived ileal organoids (IOs) to answer the following questions: (1) What are the different chemokines produced by human ileal epithelium in the absence of in vivo factors? (2) Does ex vivo chemokine secretion from the intestinal epithelium differ in composition or levels between CD and non-IBD individuals? (3) If so, are there any correlations between the levels of chemokines that are secreted by the epithelium?

Using the Luminex xMAP technology, we analyzed 30 different chemokines from the CC and CXC chemokine subfamilies (20 CC chemokines and 10 CXC chemokines) (Figure 1A). Cysteine residues closer to the N terminus are adjacent to each other in CC chemokines while CXC chemokines are characterized with a variable amino acid in between the cysteines. Chemokines were analyzed in 3-day IO-conditioned media, taken from IO cultures (Passage 2–10) from the mucosal biopsies of patients with CD (n = 8) or non-IBD control individuals (n = 6). Our results indicate that IOs secrete distinct chemokines at unique levels, and we have hierarchically organized the chemokines based on their abundance in the conditioned media (Figure 1B). Compared with negative control media, we categorized chemokine expression patterns as (1) high (above 25-fold), (2) medium (3- to 10-fold), and (3) low/none (below 3-fold). CCL24, CXCL16, CCL20, CXCL1, CCL2, CXCL4, and CCL25 were secreted at high levels (Figure 1B). Of these, CXCL4 and CCL2 are not statistically significant, likely due to the heterogeneity often observed between patient samples. Chemokines that were detected at medium levels are CCL7, CCL28, CXCL14, CXCL11, CXCL6, CX3CL1, CCL21, and CXCL2 (Figure 1B). We also observed that 14 of the 30 chemokines assayed were either secreted at very low levels or undetectable (Figure 1B). Importantly, we did not observe any significant difference in chemokine secretion levels in IO-conditioned media on average between the CD and non-IBD groups (Figure 1B). Previous studies have shown that patients with CD have elevated levels of chemokines and other cytokines in plasma.^6,7^ However, in those studies, the specific cell types involved in producing the chemokines were unclear but thought to be originating from nonepithelial cells. Our results add to the previous findings by showing the ability of ileal epithelium to produce several chemokines involved in immune cell homing and further characterizes the epithelium-specific secretome. These mucosal signals from the epithelium are likely leaked into the periphery during CD where they also act on immune cell trafficking. Remarkably, the top 3 most abundant chemokines we detected in the IO-conditioned media have been shown to recruit a variety of immune cells. CCL24 (eotaxin-2) binds to CCR3 and is implicated in the trafficking of eosinophils,^8^ CXCL16 binds to CXCR6 and plays a role in the recruitment of innate and adaptive immune cells,^9^ and CCL20 binds to CCR6 and recruits regulatory T cells, Th17, B-cells, and immature dendritic cells to the gut mucosa. Thus, the intestinal epithelium appears to play a key role in contributing to the complex repertoire of secretome products involved in immune cell recruitment to the mucosa.

Next, we determined the correlation pattern of chemokines among each other using a correlation matrix. In this approach, concentrations of each chemokine are subjected to linear regression analysis, and the correlation coefficient values (r = 1 through r = 0 implies the best to no correlation, respectively) represent the degree of their correlation (Figure 2A). One hundred and five combinations were tested among 15 chemokines showing more than 3-fold secretion levels than the negative control media (Figure 2A). We identified at least 9 best correlations among these chemokines with the r value above 0.7: CCL24 and CCL7 (r = 0.93; *P* < .0001), CXCL16 and CXCL6 (r = 0.79; *P* = .0008), CXCL16 and CX3CL1 (r = 0.79; *P* = .0008), CCL20 and CXCL11 (r = 0.80; *P* = .0005), CCL20 and CCL21 (r = 0.87; *P* < .0001), CXCL1 and CXCL4 (r = 0.82; *P* < .0001), CXCL1 and CXCL14 (r = 0.99; *P* < .0001), CXCL4 and CXCL14 (r = 0.78; *P* = .0010), CXCL6 and CX3CL1 (r = 0.91; *P* < .0001) (Figure 2B). These correlative expressions indicate that chemokines can cooperatively recruit a diverse repertoire of immune cells to the gut. For example, correlative expression between CCL24 and CCL7 suggests that eosinophils and neutrophils can be recruited by CCL24 while CCL7 can attract several leukocytes including T cells.

Taken together, the data from our novel chemokine survey of conditioned media from IO ex vivo cultures indicate that at least some of the chemokine signaling pathways in the mucosa can be mapped back to the epithelium, give new possibilities for explaining the different paths to the pathogenesis of CD when they are disrupted, and give new targets to consider for a multi-targeted therapeutic approach, where the targeting of a single molecule has often proved unsuccessful.^10^ The surprising finding here, as with our previous report, is the lack of differences on average between the groups non-IBD and CD in their secretome products; however, many differences can be observed in the levels for each chemokine between the patients’ samples, often with large differences for a few patients. The present IO chemokinome informs complexity of the chemo-attractant signaling pathways present in the gut that originate with the epithelium and is a step forward in better understanding the secretomic repertoire of the intestinal epithelium for future experimentation to gain further mechanistic insight.

## Figures and Tables

**Figure 1.**
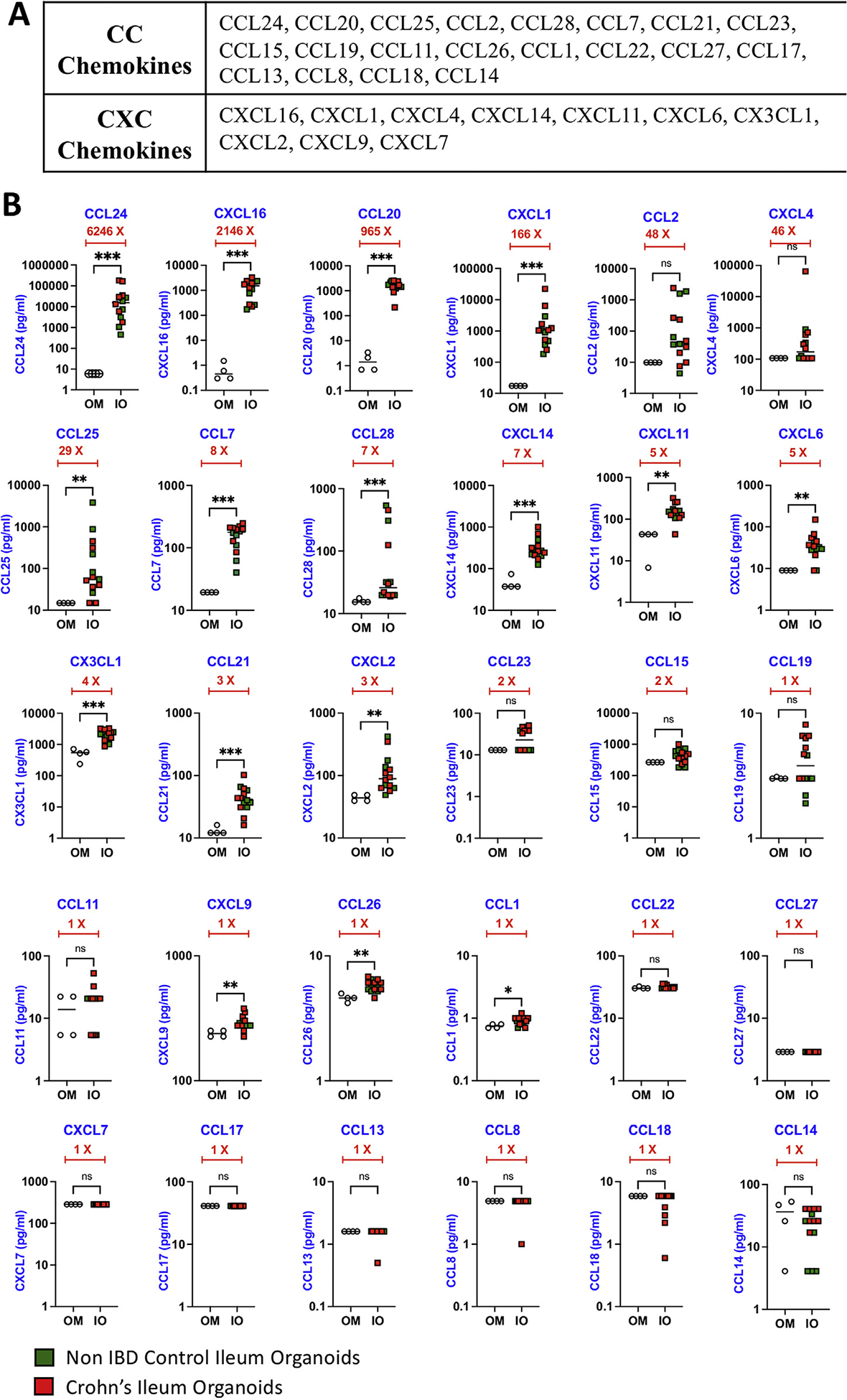
Chemokine analysis of IO supernatants. (A) List of CC and CXC chemokines tested by the Luminex xMAP technology. (B) Three-day conditioned media from organoids of non-IBD controls and CD patients was collected, cleared by centrifugation, and the supernatant used to perform multiplex analysis focusing on 30 different chemokines. Chemokine concentrations (picogram/milliliter) in each organoid culture were plotted with mean and standard deviation. Chemokine levels in fresh Intesticult media that had no previous exposure to organoids were used as a negative control. Fold change (X) in chemokine expressions between OM and IO was organized in hierarchical order of high to low difference. Two-tailed Mann-Whitney test was performed to obtain *P* values to denote the significance of mean difference. ***P < .001, **P < .01, *P < .5. ns, nonsignificant; OM, organoid medium.

**Figure 2.**
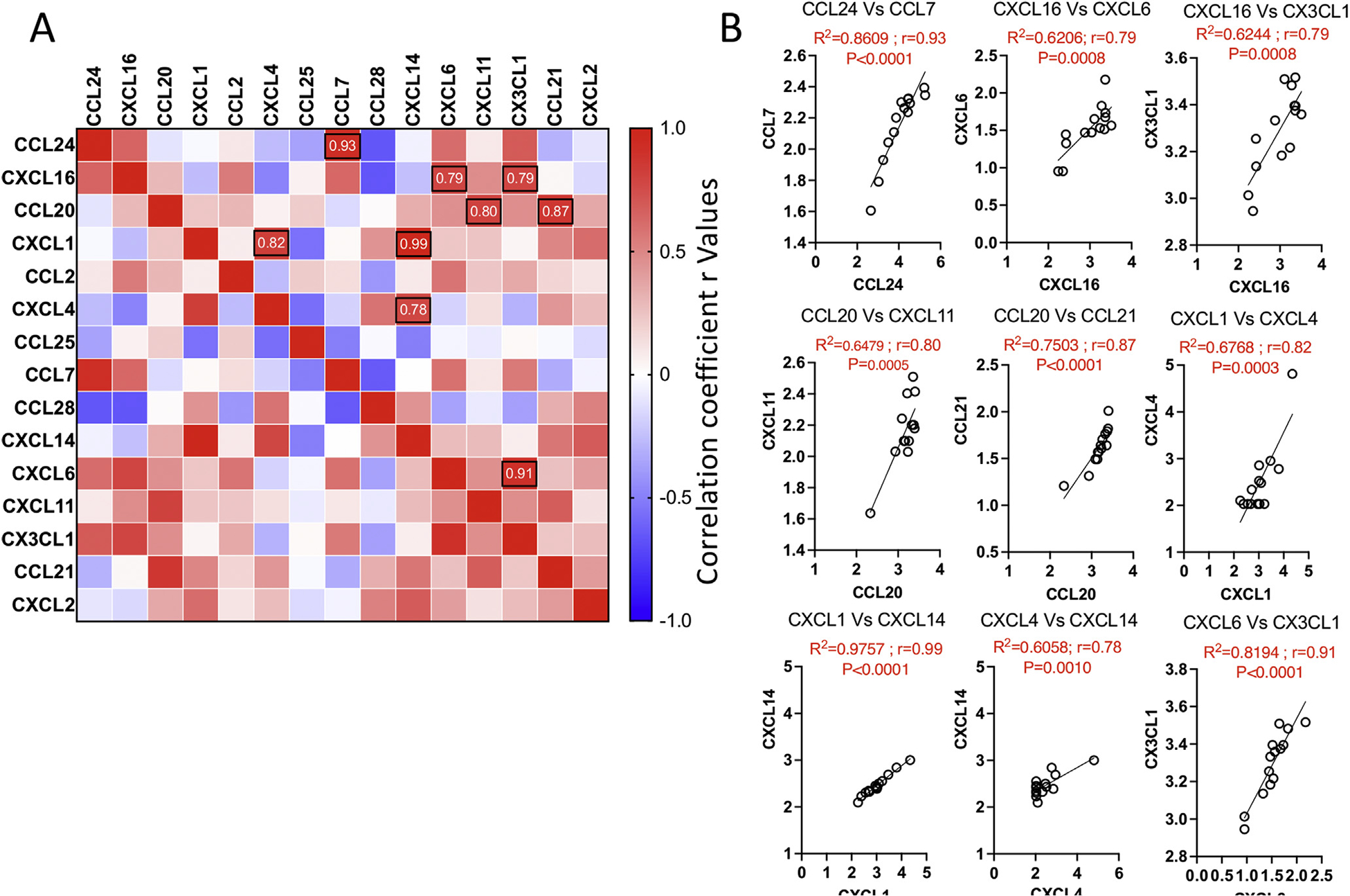
Correlation patterns among chemokines in IO cultures. (A) Individual chemokine concentrations were subjected to linear regression analysis among each other to create a correlation matrix. Pearson correlation values (r) were color-coded, and black circles indicate the best correlations based on the hierarchical ranking and above 0.7. (B) Correlative plot with Pearson correlation values (r) above 0.7 are shown. Linear regression and correlation matrix analysis was performed in GraphPad Prism to get r, R2, and *P*-values. *P*, significance of the slope deviation from zero; r, Pearson correlation coefficient values; R2, goodness of fit.

## Abbreviations used in this paper:

CD: Crohn’s disease
IBD: inflammatory bowel disease
IO: ileal organoid
